# The General Hospital, Nottingham

**Published:** 1886-12-04

**Authors:** E. M. Reely

**Affiliations:** Secretary


### THE EDITOR'S LETTER-BOX.

[Our Correspondents are reminded that prolixity is a great bar to publication, and that brevity of style and conciseness of statement greatly facilitate early publication.]

Mr. Newton H. Nixon, Secretary, University College Hospital, writes to point out that the articles, as stated on p. 144, exhibited by Messrs. Mayer and Co. were supplied by his instructions, and were exhibited by himself.

### THE GENERAL HOSPITAL, NOTTINGHAM.

May I call your attention to one or two errors which occur in your remarks in the last issue of The Hospital, respecting the Anniversary Meeting of this Institution, which took place on the 4th inst? In the first place, I may mention that Canon Morse was vicar of St. Mary's, not St. Mark's; this, however, is not of great importance as the Rev. Canon was very widely known. In the second place, I am anxious to point out to you that you have reversed the figures which relate to the expenditure of this Hospital. They should have been as follows: "In 1881-82, 1,218 in-patients and 7,224 out-patients were treated at a cost of £7,046. In 1885-86 the expenditure had fallen to £6,439, although compared with the former year 400 more in-patients and 1,500 additional out-patients received treatment." Apologising for troubling you,

E. M. Reely, Secretary.

